# Illegal Online Sexual Behavior During the COVID-19 Pandemic: A Call for Action Based on Experiences From the Ongoing Prevent It Research Study

**DOI:** 10.1007/s10508-020-01750-7

**Published:** 2020-06-02

**Authors:** Allison McMahan, Charlotte Sparre, Elin Söderquist, Stefan Arver, Gerhard Andersson, Viktor Kaldo, Katarina Görts-Öberg, Christoffer Rahm

**Affiliations:** 1 Department of Clinical Neuroscience, Centre for Psychiatry Research, Karolinska Institutet & Stockholm Health Care Services, Norra Stationsgatan 69, 113 64 Stockholm, Region Stockholm Sweden; 2 Department of Medicine, Karolinska Institutet & ANOVA, Karolinska University Hospital, Stockholm, Sweden; 3 Department of Behavioural Sciences and Learning, Linköping University, Linköping, Sweden; 4 Department of Psychology, Faculty of Health and Life Sciences, Linnaeus University, Växjö, Sweden

Due to the COVID-19 pandemic responses around the world, many people are home more often, with a range of restrictions. As a result, both children and adults have increased time spent online. Many experts have expressed concerns that this will lead to escalated online child sexual offending due to a combination of increased opportunity (ECPAT, 2020; EUROPOL, 2020; UNICEF, 2020) and heightened risk factors of stress, social isolation, and boredom of being home (Seto, 2013, 2017). Reports are also coming from the police in some countries about increases in online offending (Fitzpatrick, 2020; National Crime Agency, 2020).

In this Letter, we briefly describe “Prevent It,” an innovative online intervention for individuals who use child sexual abuse material (CSAM, previously known as child pornography), currently collecting data for evaluation of outcomes at Karolinska Institutet, and share some preliminary observations to inform fellow researchers and the public that changes have occurred during the pandemic. Based on this information, we suggest that more preventive initiatives are taken during the pandemic, specifically, in reaching out with interventions addressed directly to active online child sexual offenders.

The production, distribution, and downloading of CSAM is escalating rapidly as access to the Internet increases around the world. Recently, activity has increased across perpetrator forums on onion sites, sometimes called the darknet. The so-called onion sites apply onion services’ protocol for extra security, with the tradeoff that the users, in order to access them, need a specialized web browser such as Tor. Onion sites maintain a high degree of anonymity, hence making it difficult to connect an identity with one’s online activity. On the one hand, this allows individuals to speak freely and have more control over their privacy. On the other hand, this also makes it easier to evade law enforcement and commit illegal actions, such as the ability to download and spread CSAM with fewer ties to their identity.

**Fig. 1:**
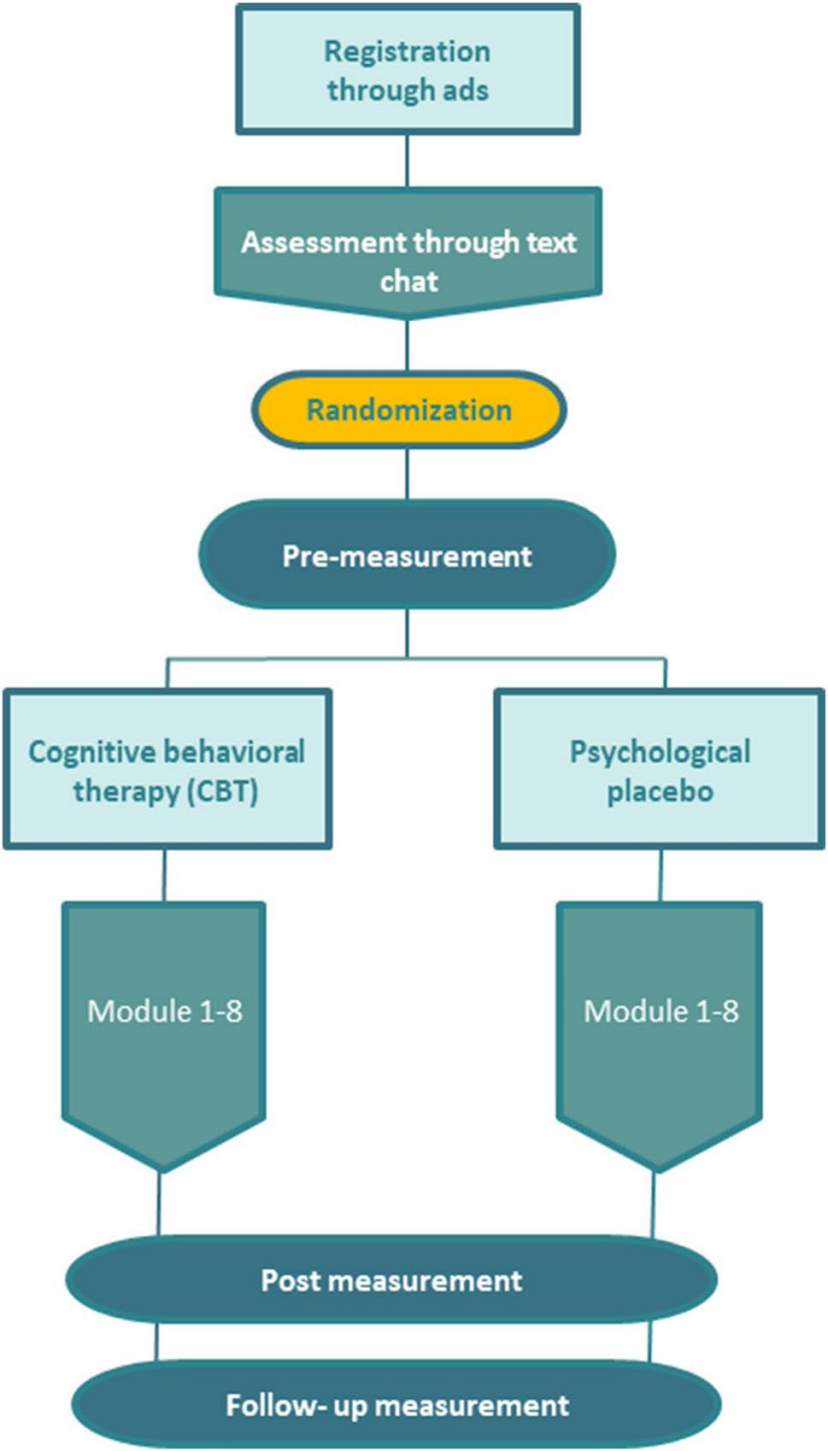
“Prevent It” study design. Participants are anonymous

“Prevent It” is an anonymous internet-based cognitive behavioral therapy (CBT) intervention which is being tested in a blinded randomized clinical trial design to see whether it is effective in decreasing consumption of CSAM (https://www.iterapi.se/sites/preventit/register). It contains weekly module content, assignments between modules, and weekly individual therapist feedback over eight weeks. It is hypothesized that CBT is more effective than an active placebo in reducing the primary outcome, which is self-reported time viewing CSAM. Secondary outcome measures include offline offending behavior, severity of CSAM, and quality of life. We believe internet-assisted psychotherapy can be effective for this patient group, as it has been shown to be as effective as in-person therapy for many other psychiatric ailments (Andersson, Cuijpers, Carlbring, Riper, & Hedman, 2014). The study is preregistered (http://www.isrctn.com/), is approved by the Swedish Ethics Appeal Review Board (Reference No.: Dnr Ö 1-2019), and its design is outlined in Fig. 1.

The target group for “Prevent It” consists of participants who currently access CSAM over onion sites and who would like to stop. Participants must be over 18 years, have accessed CSAM in the week prior to the study’s intake interview, have about one hour a week over eight weeks to dedicate to treatment, and be able to read and write confidently in English.

Recruitment for “Prevent It” occurs over specific forums on onion sites where CSAM content is discussed and linked to. Treatment is also offered through an onion site (https://6wvybf7ub3xk5ow66wt7os3aovbzoo2eei6vjirvhvvkmqg4alnezzid.onion/sites/preventit/register) to increase participant comfort about their interactions, given their concerns about privacy and possibly being identified as engaging in illegal behavior. The anonymity gives individuals an opportunity to seek care and be more open and truthful than they might be with an in-person therapist.

Recruitment began in April 2019. In each forum, a post would be made describing the study and its aims. This allowed the recruiter to start a conversation and answer questions about held views, platform security, and fears. Initially, posts were met mostly with curiosity and support; however, there have been recent changes in these online interactions.

Observations during the COVID-19 pandemic for “Prevent It” are consistent with expert concerns and other initial data suggesting online offending has increased. We have noticed the following changes in the forums: Firstly, an increased number of chatters have been seen in the forums. This has seemingly not impacted the pace of recruitment, but it is difficult to draw conclusions. In the largest forum, there are normally 100–150 active persons at one time, but after the lockdown the numbers increased to 300–400 on average. This change in activity was also seen in smaller forums. Over the last year of recruitment for “Prevent It,” there has been some variation in the patterns of posting times and activity of users noticed, and there has been a distinct change. Based on conversations of the individuals in the chat rooms, the impression is that the increase is a combination of current users having more time to spend online, new users taking the step to explore onion sites to find more extreme material, as well as users who have tried to quit but have now returned. Secondly, there has been an increase in aggressive messages, both toward other CSAM users and “Prevent It” recruiting staff. Thirdly, a change was noted in direct comments about the pandemic. Many users discussed the opportunities that could come with the changes, with comments about home schooling and babysitting during the lockdowns. Some also expressed an increased boredom and preoccupation of sexual thoughts. Lastly, a change was seen in the type of content individuals posted. There has been an increase in posts pertaining to instructions on how to get access to children to produce and share more material. These observed changes have also been confirmed with Swedish Police authority (L. Larsson, personal email communication, April 24, 2020).

Even before the pandemic, CSAM users were very active on these forums. Based on the first 40 “Prevent It” participants, the mean time spent viewing CSAM the week before registering for the study was 7.3 h per week (SD = 7.0), the mean lowest age of the children in the material was 6.2 years old (SD = 3.4), and the mean value on the COPINE scale, which measures CSAM severity on a scale from 1 to 10, from indicative material to material with sadism or bestiality, was 7.9 (SD = 1.8), which represents the higher parts of the scale. With the current work and study from home directives, it is possible these numbers have increased. While there is a greater risk for sexual offending, there is also an increased opportunity for online intervention because individuals now have more time to dedicate toward treatment if they have the motivation.

Given that pandemic responses will last months, even years, until evidence of widespread immunity or development of a vaccine, increased online offending can be anticipated, and a sustainable solution is required. More resources are needed as part of a comprehensive response that includes parent-focused interventions, child safety education, technological deterrence, law enforcement, and help for people who use CSAM or are at risk.

We are particularly interested in prevention efforts, including helplines, self-help resources, and “Prevent It” as a new option.
